# Effects of adhesive systems at different temperatures on the shear bond strength of orthodontic brackets

**DOI:** 10.15171/joddd.2019.016

**Published:** 2019-08-14

**Authors:** Serdar Akarsu, Suleyman Kutalmış Buyuk, Ahmet Serkan Kucukekenci

**Affiliations:** ^1^Department of Restorative Dentistry, Faculty of Dentistry, Ordu University, Ordu, Turkey; ^2^Department of Orthodontics, Faculty of Dentistry, Ordu University, Ordu, Turkey; ^3^Department of Prosthodontics, Faculty of Dentistry, Ordu University, Ordu, Turkey

**Keywords:** Shear bond strength, orthodontics, temperature, orthodontic adhesive resin

## Abstract

***Background.*** The temperature might affect the physical and mechanical properties of adhesive materials by reducing the polymerization rate. The present study aimed to evaluate the effect of temperature on the shear bond strength of metallic orthodontic brackets using various adhesive resin systems.

***Methods.*** Extracted human premolar teeth were randomly assigned to 8 groups (n=10) for bonding with the two available orthodontics adhesive systems (Transbond XT and NeoBond) at different temperatures: refrigeration temperature (4°C), room temperature (20°C), human body temperature (36°C) and high temperature (55°C). The shear bond strength (SBS) test was performed using a universal testing machine at a crosshead speed of 0.5 mm/min. The adhesive remnant index (ARI) was assigned to the fractured orthodontic brackets. Data were analyzed with one-way ANOVA, post hoc Tukey tests and independent t-test.

***Results.*** Transbond XT exhibited higher SBS values compared to Neobond at all the tested temperatures; however, a statistically significant difference was not observed (P>0.05). The SBS results were minimum at 4°C and maximum at 36°C in both the adhesive groups (P<0.05).

***Conclusion.*** Pre-heating orthodontic adhesives up to the body temperature prior to bonding the brackets in orthodontic treatment increased the bond strength of orthodontic brackets.

## Introduction

Buonocore^1^ introduced acid etching technique, thereby revolutionizing the dental practice. Thus, a strong connection was established between dental tissues and developments in the restoration procedures in the adhesive systems in 1955. Newman first applied acid etching in orthodontics in 1965.^2^ The adhesion of orthodontic brackets used in fixed orthodontic treatment to the tooth and the strong bond between the tooth and the bracket is one of the factors affecting the success of orthodontic treatment. For an effective orthodontic treatment with fixed appliances, adequate bonding between the enamel surface of the tooth and the bracket is essential. Failure in bonding the brackets to the enamel surface reduces the success of orthodontic procedures while increasing the duration and cost of treatment.^3^

Briefly, roughening the enamel with 37% phosphoric acid is the first step in direct bonding of brackets in orthodontic treatment. This process allows formation of a prism crown on the enamel, thereby allowing the penetration of the resin and ensuring an effective mechanical lock.^4^

Several factors affect the bond strength of orthodontic brackets, including contamination, type of composite resin, viscosity of the adhesive, age of the composite resin, etching type of enamel, storage conditions, size and shape of the bracket base, and type of the test used.^5-9^ Furthermore, temperature and humidity are critical factors.^10^ Although the majority of manufacturers recommend that adhesive materials be stored at room temperature, these materials are cooled to extend the shelf life, and in clinical practice, clinicians use the material from a refrigerator without allowing it to reach the room temperature. Intriguingly, low temperature can lead to a decrease in the effectiveness of the adhesive material. The changes in the waiting temperature of the adhesive material might adversely affect the physical and mechanical properties of this material by reducing polymerization.^11^

Resin materials cannot be fully polymerized when used at room temperature^12^ and when insufficient energy is received by light activation.^13^ Friedman described a polymerization method, wherein a high degree of monomer conversion was obtained by heating the composite resin to 54–60°C prior to the activation of light.^14^ Pre-heating can lead to an increase in polymerization depth^15^ and molecular mobility.^16,17^ As a result, polymer chains multiply and polymerization is optimized.

Therefore, the present study aimed to evaluate the effect of temperature on the shear bond strength of different adhesive resins used for bonding brackets to the enamel in orthodontic treatment.

## Methods

### Preparation of Samples

The present cross-sectional in vitro study was performed in the Dentistry Faculty, Ordu University (Ordu, Turkey). The study was approved by the Local Clinical Research Ethics Committee of the Ordu University (2019-32). The sample size was calculated based on a power analysis using G*Power Software version 3.1.9.2 (Universität Düsseldorf, Germany) for shear bond strength at alpha error probability of 0.05 and a power of 95%.^18^ The power analysis showed that a total of 10 samples (effect size = 3.375645) was enough, while reliable results could be determined using 80 premolar teeth. Subsequently, 80 freshly extracted premolars for orthodontic reasons were used in this in vitro study. The teeth were cleaned and polished with non-fluorinated pumice. Non-carious and non-cracked teeth on the buccal enamel surface were included in the study.^19^ Premolar teeth were randomly assigned to 8 groups (n=10) for bonding with two available orthodontic adhesive systems at various temperatures: refrigerated temperature (4°C), room temperature (20°C), human body temperature (36°C), and high temperature (55°C).

**Transbond XT Group:** The teeth were rinsed with 35% phosphoric acid for 30 seconds using a syringe, washed and dried. Then the Transbond XT Primer (3M Unitek, Monrovia, CA, USA) was applied, followed by air jet and curing for 3 seconds.

**NeoBond Group:** The teeth were subjected to acid treatment as above. Then, the NeoBond primer (Dentsply DeTrey; Konstanz, Germany) was applied on the teeth.

Orthodontic metal brackets (0.022×0.028-inch, Mini Master, American Orthodontics, Sheboygan, Wisc) were used in all the groups. A standardized stable 150-g force was applied to the top surface of the metal brackets using a tension gauge (Correx, Haag-Striet, Bern, Switzerland) to obtain a uniform adhesive thickness. Subsequently, the excess adhesive was removed, and curing was performed for 6 seconds/tooth and 3 seconds on each proximal face, with a light-curing unit (Valo Ortho LED, Ultradent Products Inc., South Jordan, USA). The bonded specimens were preserved in deionized water at 37°C for 24 hours.^18^

### Shear Bond Strength (SBS) Test

**Figure 1:**
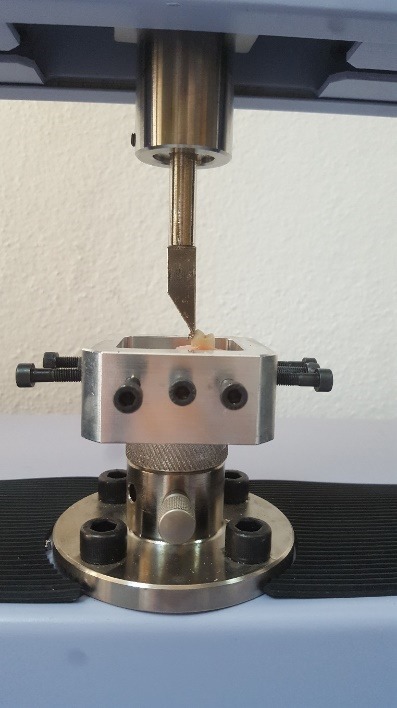
The design of experimental shear bond strength test of orthodontic brackets.

The shear bond strength (SBS) test was performed using a universal testing machine (Shimadzu Instron, Shimatsu Corp., Kyoto, Japan) at a crosshead speed of 0.5 mm/min. The shearing wedge was positioned vertically at the bracket base (Figure 1).^15^ The SBS data were expressed in MPa.

**Figure 2:**
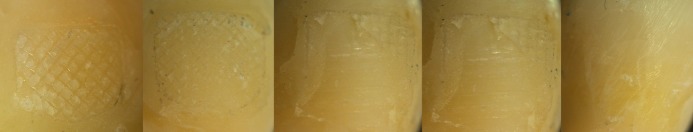
Adhesive remaining on different tooth surfaces after debonding

After debonding, the brackets were examined under ×10 magnification,^19^ and the ARI was defined as follows: 5: no adhesive paste remaining on the enamel surface; 4: <10% of the adhesive paste remaining, 3: >10% but <90% of the adhesive paste remaining; 2: >90% remaining; 1: all the adhesive paste, with an impression of the bracket base, remaining (Figure 2).^19^

### Statistical Analysis

Statistical analysis (SPSS 20.0; SPSS Inc, Chicago, IL, USA) of the SBS (MPa) data was performed using one-way ANOVA and post hoc Tukey tests on the normally distributed data (Shapiro–Wilks test). The frequency distribution of the ARI scores among the groups was evaluated using chi-squared test. Statistical significance was defined at P<0.05.

## Results

The results of the SBS evaluation are shown in Table 1. While the SBS values of Transbond XT were between 10.29 and 16.15 MPa, those of Neobond were between 8.66 and 15.30 MPa. Thus, it was observed that Transbond XT had higher SBS values than Neobond at all the temperatures, albeit without any statistically significant differences (P>0.05). In both groups, SBS results at 4°C were the lowest, while those at 36°C were the highest amongst all the temperatures tested in the present study, with a statistically significant difference (P<0.05). The results of ARI evaluation are shown in Table 2. None of the specimens showed any rupture in the enamel; a statistically significant difference was not detected between the groups.

**Table 1:** Shear bond strength (MPa) of two adhesive orthodontic bracket bonding systems at different temperatures

***Temperature***	***Transbond XT***	***NeoBond***	***P*** **-v** ***alue***
	**Mean (SD)**	**Mean (SD)**	
***4°C***	10.29 (2.91) A	8.66 (1.96) a	.159^α^
***20°C***	12.91 (2.84) A	11.06 (2.83) a	.163^α^
***36°C***	16.15 (1.68) B	15.30 (3.76) b	.525^α^
***55°C***	12.70 (2.22) A	10.80 (2.30) a	.077^α^
***P-value***	.000^β^	.000^β^	

^α^Results of independent t-test; ^β^, Results of one-way analysis of variance test; SD, standard deviation. Groups with different uppercase or lowercase letter are significantly different (Tukey HSD test, P<0.05).

**Table 2:** Distribution of adhesive remnant index (ARI) scores of the debonded brackets

		**ARI scores**				
		**1**	**2**	**3**	**4**	**5**
**Transbond XT**	**4°C**	2	1	4	2	1
	**20°C**	1	5	2	1	1
	**36°C**	1	2	5	1	1
	**55°C**	1	4	2	1	2
**NeoBond**	**4°C**	1	2	3	2	2
	**20°C**	-	4	1	3	2
	**36°C**	1	4	2	2	1
	**55°C**	2	3	4	1	-

## Discussion

Although some investigators claim that the bond strength of 2.86 MPa is clinically acceptable, the minimum bond strength for orthodontic brackets is 6–8 MPa.^6,7,9,20^ The results of the current study revealed that the two adhesive systems applied at different temperatures showed sufficient bond strength at 8.66–16.15 MPa. Reportedly, the adhesive temperature does not significantly affect the bond strength of tooth structures;^21,22^ however, some studies demonstrate that the temperature affects the adhesive strength of the adhesive.^23-25^ In the present study, the minimal bond strength values were obtained at 4°C. The adhesive viscosity increases significantly at low temperatures,^26,27^ which makes it difficult to wet the dental tissue due to a decrease in the adhesive speed.^28^ Herein, the preheated adhesive systems up to 36°C showed higher SBS values than the lower temperature adhesive systems. This increase in SBS values can be explained by the decrease in viscosity, the increase in radical mobility, and the degree of transformation.^29^

Furthermore, when the adhesive systems used in this study were heated up to 55°C, the SBS values were found to be lower than those obtained at 36°C. Some components of adhesives, such as HEMA, MDP and BIS-GMA, can be chemically deteriorated due to their unsuitability for high temperature.^30^ As a result, the bond strength of the adhesive might decrease.

Parameters such as shear and tensile strengths are used to measure the resistance of the teeth. However, the disadvantage of these methods is that they are usually applied on flat surfaces. In these cases, the C factor is low, and the shrinkage stress is not directed to the bonding interface.^31^

Previous studies demonstrated that the type of the adhesive material, the in vitro test used and the adhesive thickness affect the bond strength of orthodontic adhesives.^32^ Muguruma et al^33^ reported that the application of a force >200 g during the bonding procedure of the brackets is preferred to obtain a thin composite resin layer and to ensure sufficient adhesion of the adhesive.^34^ However, in the current study, a 150-g force was applied using a force gauge to bond the brackets and optimize the thickness of the adhesive.

In the orthodontic treatment, the brackets are removed by the failure in one of the three interfaces on the tooth surface. No adhesive remained on the tooth surface in the adhesive failure; however, adhesive material was present on the tooth surface and the bracket base in cohesive failure.^35^ The adhesive failure occurs in the adhesive cement and between the tooth surface and the adhesive cement, while the cohesive failure occurs between the adhesive cement and the bracket. It is recommended that the bond between the bracket and the tooth structure should be sufficiently high to resist the mechanical forces applied by the physician, the chewing and the parafunctional forces, while the integrity of the enamel should be maintained during debonding.^36^ When the adhesive remains on the enamel surface after debonding due to cohesive failure, the enamel might be damaged, increasing the duration of the procedure.^37^ Therefore, cohesive failure is not favorable. Subsequently, the ARI scores of this study were evaluated, and no differences were detected between the body temperature and the other temperatures with respect to the highest SBS results. In 76% of the samples, an adhesive residue persisted on both the enamel and the bracket surface. None of the specimens showed a failure in the enamel after the test. Faria-Júnior et al^38^ established a direct correlation between ARI scores and SBS. High SBS value is associated with a high ARI score. In the present study, the ARI scores corresponding to the highest bonding strength value in some groups were found to be different, rendering the prediction of the failure type challenging for a specific bonding force. Thus, the ARI scores and bonding strength values might not be compatible with each other.

## Conclusion

NeoBond, a fluoride-releasing orthodontic adhesive, works in a manner similar to Transbond XT, which has been used for several years in bonding brackets in orthodontic treatment. Therefore, it might be effective in preventing enamel calcifications and enamel caries that occur in long-term orthodontic treatments.

Although most manufacturers recommend that the adhesive systems used in dentistry be stored in a refrigerator, pre-heating of the adhesives to body temperature before bonding the brackets in the orthodontic treatment increases the adhesion of the brackets to the enamel.'

## Authors’ contributions

The concept and the design of the study were developed by SA and SKB. The experimental design of study was performed by SA, SKB and ASK. Statistical analyses were carried out by SKB. The manuscript was written by SA and SKB. The proof reading was carried out by SA and SKB. All the authors participated in the literature review.

## Acknowledgments

None.

## Funding

None.

## Competing interests

The authors declare that they have no conflict of interests.

## Ethics approval

The study was approved by the Local Clinical Research Ethics Committee of the Ordu University, Turkey (2019-32).

## Informed Consent

Written informed consent was obtained from the volunteers who participated in this study.

## Conflict of Interest

The authors have no conflict of interest to declare.

## Financial Disclosure

The authors declared that this study has received no financial support.
